# CuAgent provides a RAG-assisted intelligent framework to investigate cuproptosis

**DOI:** 10.1038/s41598-026-49929-7

**Published:** 2026-04-22

**Authors:** Chunlong Zhang, Jin Bao, Haojie Yu, Haijie Cui, Xuecang Li, Jianli Ma, Ning Zhao

**Affiliations:** 1 College of Computer and Control Engineering, Northeast Forestry University, Harbin, 150040 China; 2 Department of Radiation Oncology, Harbin Medical University Cancer Hospital, Harbin, 150081 Heilongjiang China; 3 School of Medical Informatics, Daqing Campus, Harbin Medical University, Daqing, 163000 China

**Keywords:** Cuproptosis, Intelligent agent, Natural language processing, Retrieval-augmented generation, Bioinformatics platform, Cancer, Computational biology and bioinformatics

## Abstract

Cuproptosis is a novel form of regulated cell death driven by intracellular copper accumulation, leading to lipoylated protein aggregation and Fe-S cluster destabilization. Dysregulation of this process has been implicated in various pathological conditions, including cancers, neurodegenerative diseases and metabolic diseases. Despite rapidly growing interest in cuproptosis, a systematically curated intelligent agent dedicated to cuproptosis-related genes (CRGs) and their disease associations remains lacking. To address this, we constructed a cuproptosis-related artificial intelligence (AI) knowledge base, named CuAgent, by manually curating 465 experimentally validated CRGs and 163 associated diseases. CuAgent introduces an innovative intelligent agent that enables users to perform natural language queries and receive data-driven responses. In addition, agent offers gene queries and analytical tools (expression profiling, survival analysis, protein interaction network visualization and correlation analysis). This study provides critical insights into cuproptosis progression, presenting a comprehensive and interactive resource to advance the understanding of cuproptosis. URL: https://bioinfor.nefu.edu.cn/CuAgent/home/.

## Introduction

Cuproptosis is a recently identified form of copper-dependent regulated cell death, characterized by molecular mechanisms distinct from other known types of cell death such as apoptosis, necroptosis and ferroptosis^1,2^. The process is driven by specific binding of copper ions to lipoylated components within mitochondrial tricarboxylic acid TCA cycle, which induces abnormal aggregation of key metabolic enzymes and promotes destabilization of iron-sulfur cluster proteins^3^. These events culminate in severe proteotoxic stress and ultimately result in irreversible cell death.

Cuproptosis dysregulation has been increasingly implicated in the pathogenesis of diverse complex diseases. CRGs are critical modulators in tumor microenvironment^4,5^. For instance, low expression of *FDX1* in kidney renal clear cell carcinoma (KIRC) was linked to tumor progression and poor prognosis. PCR, Western blot and Immunohistochemical analysis showed *FDX1* knockdown promoted cell proliferation and invasion, with lower expression in tumor tissues compared to normal kidney^6–9^. *FDX1* expression was reduced in hepatocellular carcinoma, with higher levels linked to improved prognosis, likely by influencing copper metabolism and cuproptosis to hinder tumor growth. Experiments revealed that *FDX1* overexpression decreased proliferation and migration, as shown by CCK-8 and transwell assays, and flow cytometry and western blot indicated increased apoptosis and alterations in associated proteins^10^. The expression levels of *LIAS* and *LIPT1* were significantly linked to patient survival. Immunohistochemistry revealed marked expression differences in gastric cancer. These genes can serve as biomarkers for cuproptosis-related subtypes, aiding in gastric cancer molecular subtype classification^11^. In contrast, immunohistochemistry confirmed that *DLAT* was highly expressed in lung and colorectal cancer and is closely associated with prognosis^12^.

Since the initial delineation of its molecular mechanisms in 2022, research on cuproptosis has expanded rapidly. As of May 2025, over 1,755 articles related to cuproptosis have been indexed in PubMed, marking a substantial increase from earlier sporadic reports. Nevertheless, the field continues to face significant challenges, such as the absence of comprehensively integrated and experimentally validated datasets and the lack of a standardized framework for biomarker evaluation. These limitations considerably hinder the clinical translation of cuproptosis-related findings.

The rapid development of artificial intelligence (AI) is profoundly transforming biomedical research, particularly in understanding complex disease mechanisms and identifying potential therapeutic targets. Among these innovations, the integration of large language models (LLMs) and Retrieval-Augmented Generation (RAG) has become a powerful tool for leveraging vast biomedical literature, predicting gene-disease relationships and extracting valuable insights from high-dimensional omics data^13–16^. BiomedRAG significantly enhanced performance in information extraction, text classification and question answering by directly incorporating retrieved document chunks into LLM^17,18^. A dual-retrieval and ranking framework combined semantic search with Elasticsearch, improving accuracy in complex medical queries while maintaining real-time responsiveness^19^. Furthermore, domain-specific ontologies like MIO emphasized the crucial role of structured knowledge in supporting precision medicine research^20,21^. These advancements demonstrated the significant value of RAG in processing large-scale biomedical data, advancing knowledge generation and facilitating practical applications in the field.

**Fig. 1:**
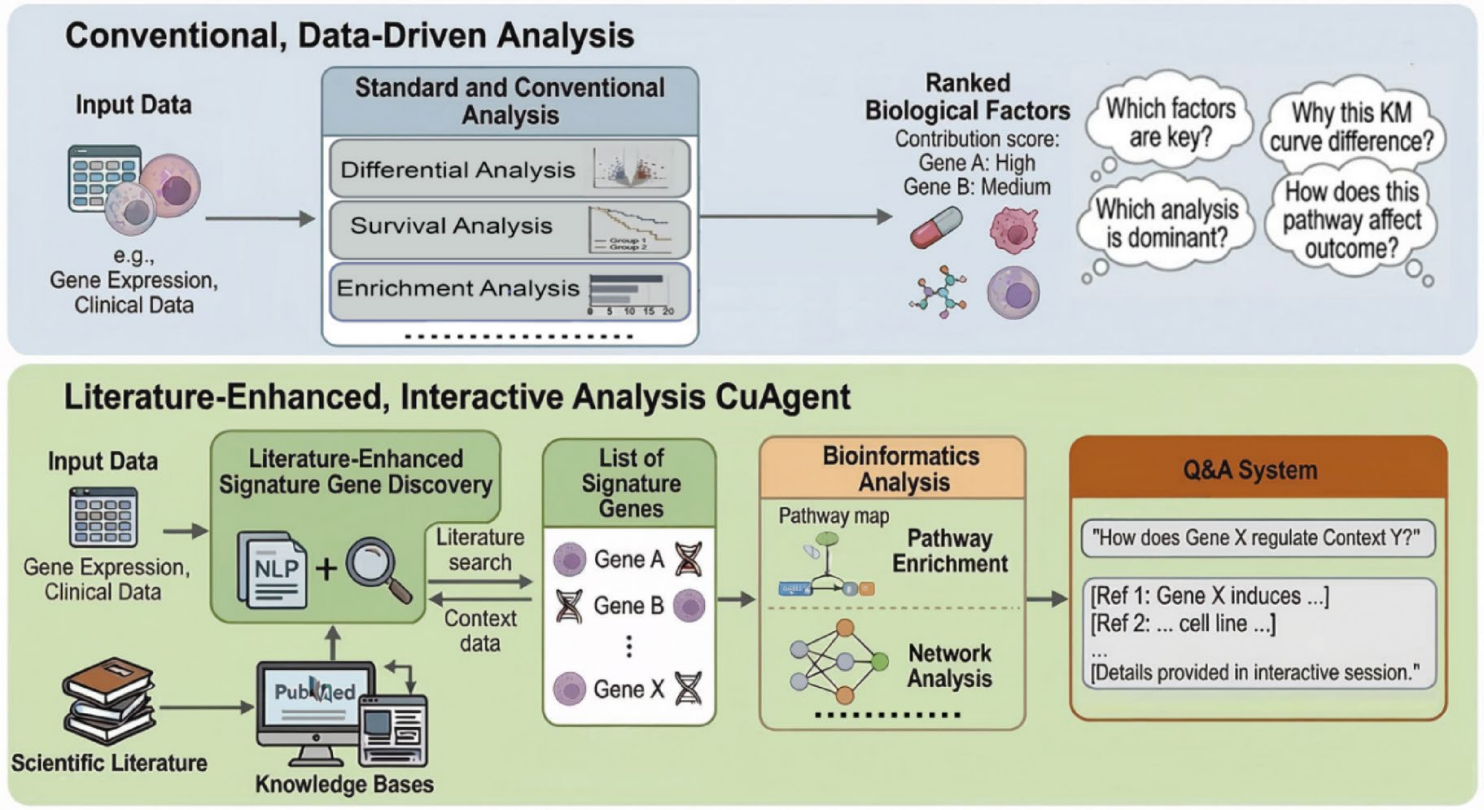
The comparison between CuAgent and conventional analysis.

To address these gaps, we developed CuAgent, a RAG-assisted intelligent framework for cuproptosis research (Fig. 1). Built upon a meticulously curated database of experimentally validated CRGs and their multi-dimensional disease associations, CuAgent significantly transcends conventional platforms. Beyond providing standard structured results via routine bioinformatics analyses, its core innovation lies in a natural language conversational interface that interactively delivers unstructured, literature-backed mechanistic insights. Ultimately, by integrating quantitative analysis with dynamic evidence retrieval, CuAgent serves as an efficient and reliable tool to facilitate hypothesis generation and guide future experimental designs.

## Materials and methods

### Literature collection

**Fig. 2:**
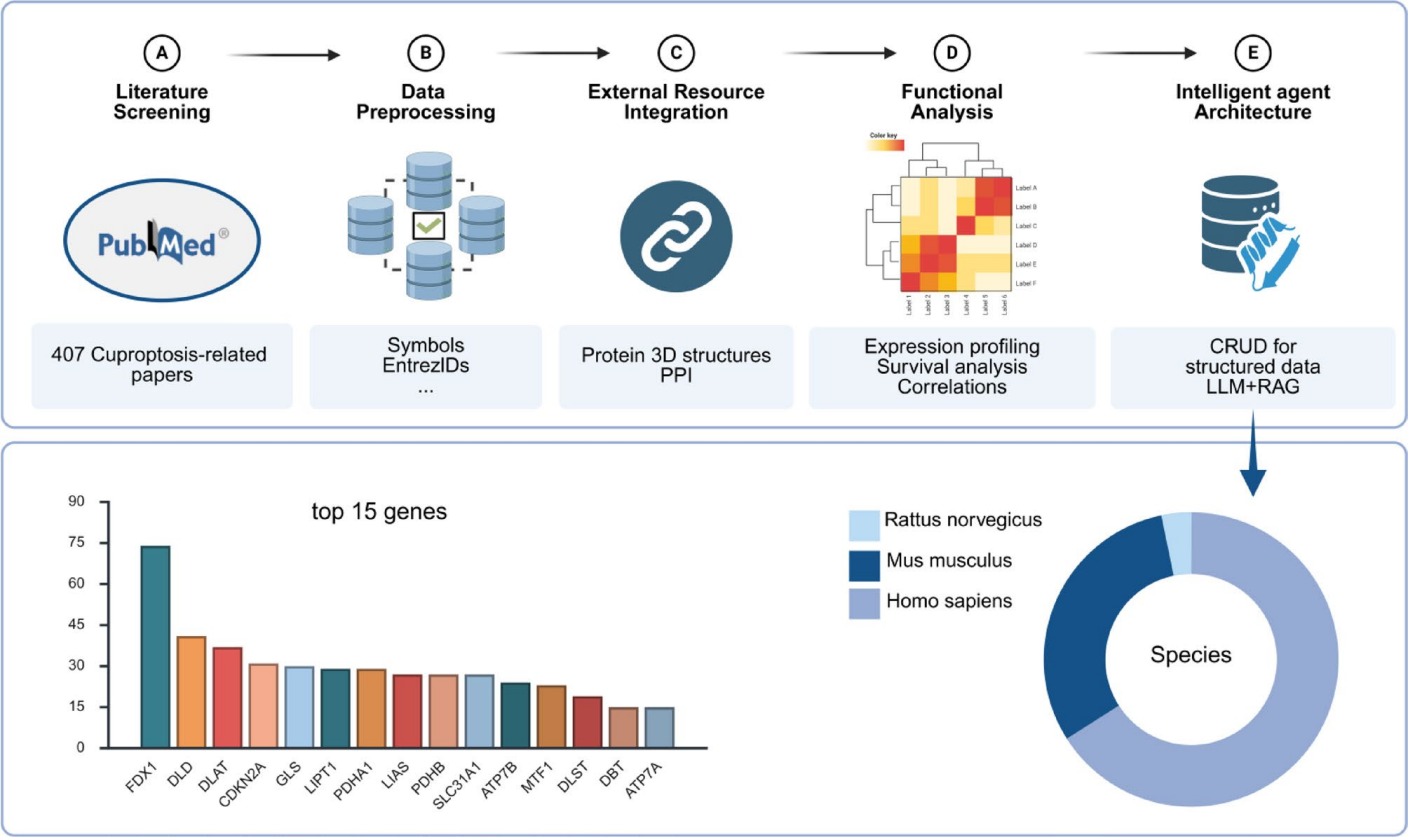
Workflow of CuAgent construction. (**A**) Literature collection. (**B**) Data preprocessing. (**C**) External resource integration. (**D**) Functional analysis module. (**E**) Intelligent agent.

To ensure the accuracy and reliability of the data, all data in CuAgent were manually curated through a systematic literature collection process (Fig. 2). By searching the PubMed (https://www.ncbi.nlm.nih.gov) with keywords “Cu”, “Cuproptosis” and “copper-induced cell death”, 1,755 articles were obtained. The literature screening was conducted based on predefined inclusion criteria. Only peer-reviewed research articles were considered. Studies were required to provide clear experimental methods, such as Western blotting, qRT-PCR and so on. Additionally, all selected studies must directly focus on the studies about CRGs. The exclusion criteria encompassed review articles, studies solely based on bioinformatic predictions, and articles for which the full text was unavailable. Data extraction was conducted in accordance with a structured framework, including gene information, experimental method, species, disease association, regulatory direction and direct quotations from the source literature (Table 1).

**Table 1:** Data extraction fields.

Field category	Specific content
Gene information	Gene name
Experimental method	Experimental method
Species	Species
Disease association	Disease name
Regulatory direction	Up-regulated/Down-regulated relative to control samples (must be explicitly stated in the source)
Experimental evidence	Direct quotations or descriptions extracted from the source literature

### Data pre-processing and text mining

To ensure data standardization and traceability, we implemented a multi-level biomedical data integration strategy for the systematic annotation of CRGs and their disease associations. Gene nomenclature was standardized according to the HGNC (v2023-10-15, https://www.genenames.org/). Cross-validation was performed using NCBI Gene (v2023-10-30, https://www.ncbi.nlm.nih.gov/gene) and Ensembl (v110, https://asia.ensembl.org/)^22,23^. Genes were simultaneously mapped against NCBI Gene and Ensembl, and were retained only if their official Symbols, Entrez IDs, Ensembl IDs, and species annotations demonstrated perfect concordance. Disease name and classification information were annotated using the Disease Ontology database (v2023-12-15, http://Disease-ontology.org) to maintain ontological consistency^24^. To ensure high reliability, we strictly included only those genes whose altered expression or functional roles were explicitly supported and experimentally validated in the original literature through methods such as Western blot, qRT-PCR, or luciferase reporter assays (*p* ≤ 0.05). Each evidence record was annotated with the specific experimental technique and the corresponding PubMed ID.

### Implementation of the intelligent Q&A agent

**Fig. 3:**
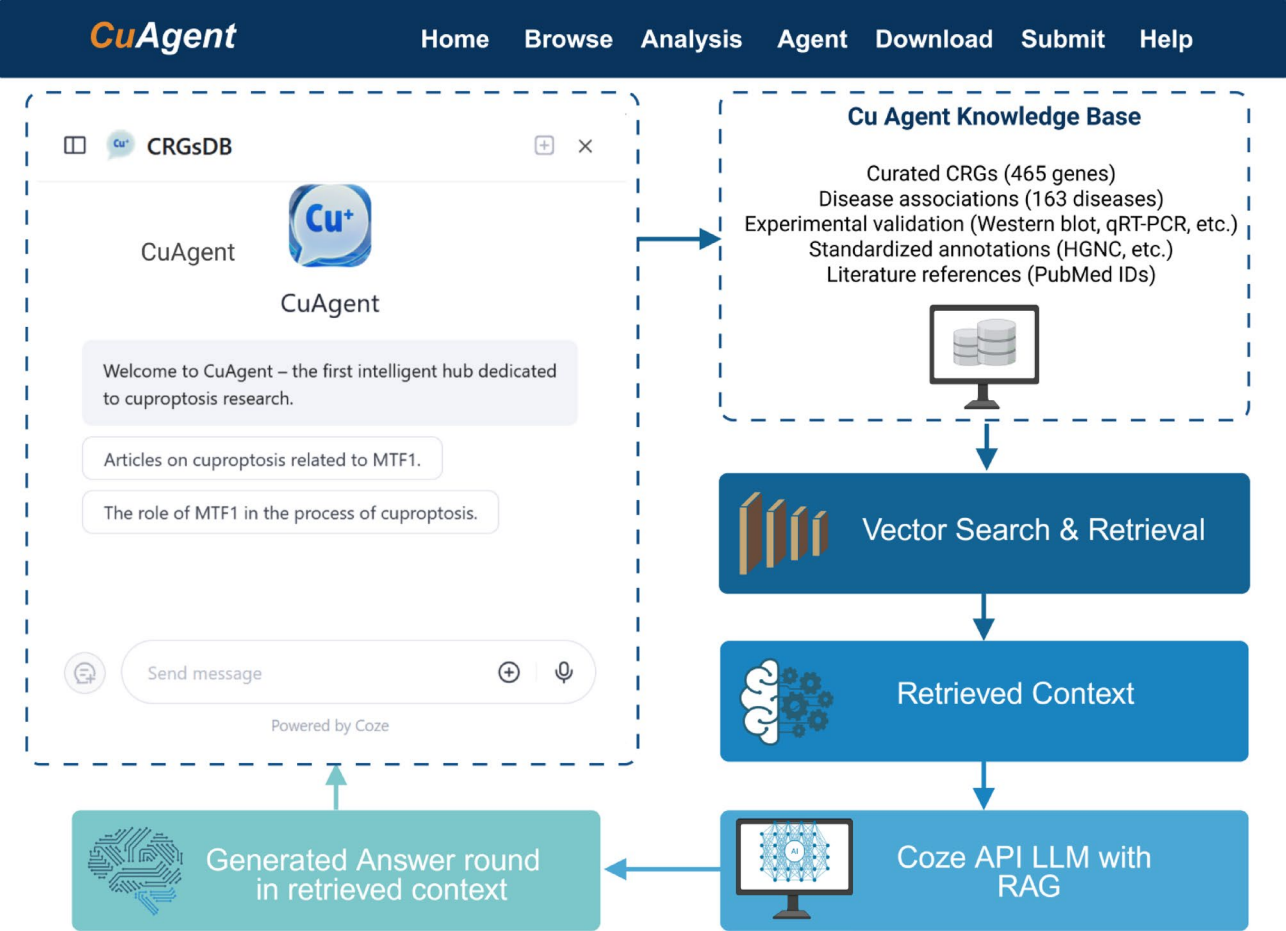
Workflow of the intelligent question-answering agent based on RAG architecture.

The intelligent agent was effectively incorporated into the CuAgent platform, providing a natural language interface that enables users to query curated knowledge concerning CRGs and their disease associations (Fig. 3). This agent facilitated dynamic and evidence-based interactions, allowing researchers to efficiently investigate complex biological relationships without being limited to traditional keyword-based search or manual browsing^25^.

To achieve strict domain adaptation without compromising the model’s generalized reasoning capabilities, we implemented a RAG framework rather than performing full-parameter fine-tuning. To overcome local computational limitations (e.g., GPU memory and processing power) during high-concurrency operations, we deployed the CuAgent platform via Coze API. This high-performance AI platform provides reliable cloud computing support, ensuring rapid response times and stability while reducing local hardware loads. We selected DeepSeek-V3.2 as our foundational model due to its outstanding capabilities in logical reasoning and instruction following^26,27^. To ensure rigorous reproducibility, we utilized the API version-locking feature provided by Coze, guaranteeing that the inference logic remains strictly consistent over time. Upon submission of a query, the system employs semantic vector search to initially interrogate a meticulously curated cuproptosis knowledge base to extract pertinent and scientifically validated information, subsequently generating a response strictly grounded in the retrieved context. This comprehensive knowledge base included standardized gene annotation, experimental finding, disease association and corresponding reference from literatures. By anchoring responses in retrieved evidence within a strictly closed-domain environment, this design ensured that all answers could be traced back to authoritative sources and specific PubMed IDs, thereby substantially mitigating the risk of model hallucinations (Fig. 3B).

To improve usability, the Q&A interface was embedded directly into the CuAgent as a perpetually accessible chat widget. System performance has been optimized for real-time interaction, with the majority of responses generated in less than three seconds. To maintain session isolation and context association during complex, multi-turn interactions, users are prompted to provide an email address. This acts purely as a unique session identifier to enable features like query tracking and API rate limiting. We strictly enforce a privacy-first policy: these identifiers are securely hashed, never used for data collection, and never shared with third parties. Users prioritizing absolute anonymity may utilize pseudonymous emails to access the full functionality as a guest.

### Resource integration

To augment the functional comprehensiveness and analytical capabilities of the CuAgent, we systematically incorporated a variety of external biological data resources, employing corresponding technical strategies.

High-accuracy predicted three-dimensional protein structures were acquired from the AlphaFold Protein Structure Database (v4,https://alphafold.ebi.ac.uk/)^28^. For transcriptomic analysis, we implemented GEPIA2 (v2, http://gepia2.cancer-pku.cn/) API interface^29^, enabling batch retrieval of gene expression profiles from the TCGA and GTEx projects^30,31^. This integration supported large-scale gene expression analysis, survival analysis and multi-gene correlation analysis. Furthermore, protein-protein interaction networks were constructed with experimentally validated and predicted interaction data from the STRING database (v11.5, https://cn.string-db.org/)^32^, which were dynamically visualized in the web interface through integration with ECharts, providing users with interactive exploration capabilities.

### System design and implementation

The Data Layer utilized a MySQL 8.0 relational database for structured data storage. Object-Relational Mapping (ORM) was implemented via the MyBatis 3.5 framework. This layer was deployed within an Apache Tomcat 9.0 application server cluster environment.The Business Logic Layer was developed using Java (v11) and the Spring Boot (v2.7) framework. The Frontend Presentation Layer was built using the Vue (v3.2) + Vite (v4.0) toolchain. Interactive tables were rendered using DataTables (v2.0), and multi-dimensional data visualization was achieved using ECharts (v5.4). The intelligent Q&A agent was powered by the Coze AI platform. Leveraging RAG technology, it combined user queries in natural language with the structured data in CuAgent in real-time to generate accurate answers supported by literature.

## Results

### Overview of the CuAgent

The CuAgent employs a modular architecture that integrates six core functional modules designed to support cuproptosis research (Fig. 3).

**Fig. 4:**
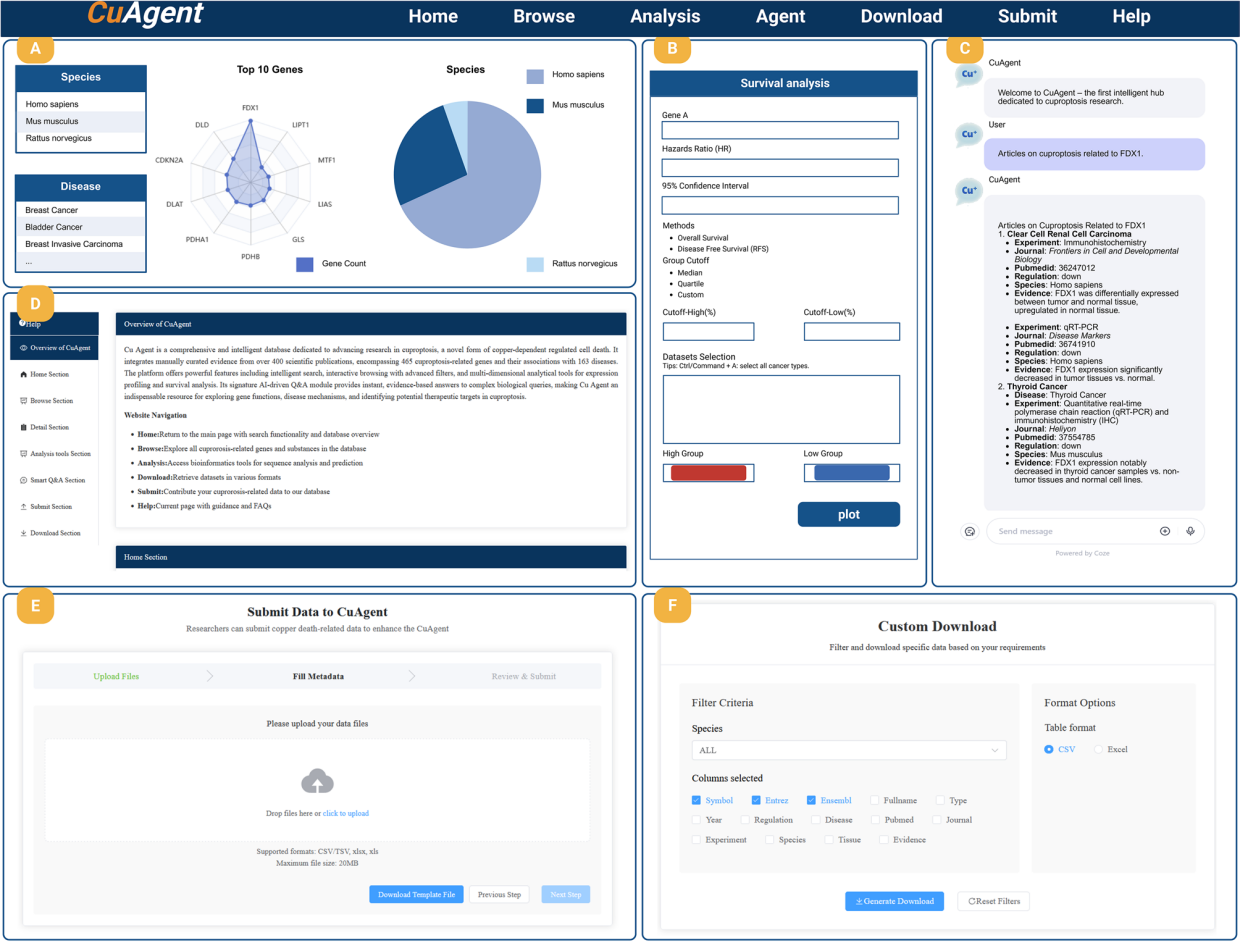
Core functional modules of the CuAgent. (**A**) Browsing interface with filtering; (**B**) Analysis tools interface; (**C**) AI Q&A agent; (**D**) User help center; (**E**) Data contribution portal; (**F**) Data download.

### Data browse

The agent provides structured query and intelligent Q&A capabilities, with structured query categorized into quick search and data browse. The quick search supports direct retrieval by gene symbol (e.g., FDX1), enabling rapid access to specific entries. Alternatively, the browse page employs a tree-view structure that visually represents hierarchical gene-disease relationships, with filtering capabilities across multiple categories including gene name, regulatory direction (up/down-regulated‌), disease information and species information.

The browsing interface incorporates a multi-level classification system that allows users to explore data based on gene function and disease type. Dynamic filtering options further facilitate cross-category exploration using combined conditions such as species (human/rat/mouse), regulatory direction (up/down-regulated‌) and disease information.

Each gene features a dedicated detail page integrating five functional modules: Basic Information (with gene identifiers and links to external databases), Protein Structure (interactive 3D visualization via AlphaFold), Experimental Evidence (direct quotes and PMIDs from literature), Integrated Analysis Toolkit (gene expression analysis, survival analysis, and correlation visualization) and an Intelligent agent.

### Intelligent agent

The intelligent agent is a core feature of CuAgent. It tightly integrates the Coze AI platform with our manually curated and high-quality knowledge base (Fig. 4C), ensuring that every response is rooted in verified and authoritative data.

Users can input gene names or pose questions in natural language—such as “What role does MTF1 play in cuproptosis?“—and receive instant, precise and evidence-backed answers. Unlike generic AI model, CuAgent. is built specifically for the cuproptosis research domain, making it exceptionally accurate and context-aware.

This system surpasses basic fact retrieval by integrating curated domain knowledge, experimental data and scientific literature to generate comprehensive and reliable insights. These insights are instrumental in shaping research hypotheses, designing experiments and validating findings. Consequently, it serves as a valuable tool for both researchers and clinicians, providing actionable information that enhances the efficiency of data retrieval and knowledge application.

### Analysis tool

To enhance the practicality of agent, we offers five integrated analytical tools (Fig. 3B): (i) Expression profile analysis visualizing gene expression levels (TPM/FPKM) across tissues using TCGA and GTEx data; (ii) Gene expression analysis identifying tumor-specific genes under customized criteria; (iii) Survival analysis employing Kaplan-Meier methodology to evaluate associations between gene expression and clinical outcomes; (iv) Interaction network construction deriving protein-protein interaction networks from the STRING database; (v) Correlation analysis calculating gene expression correlations using Pearson/Spearman methods.

### Data submit and download

To facilitate community-driven curation and ensure the continuous growth of the agent, we established a streamlined data submission mechanism (Fig. 4E). Users can contribute new findings or suggest updates through standardized online forms. Additionally, comprehensive user support is available, including interactive guides and illustrated tutorials. Moreover, the website’s data supports free download. (Fig. 4F), allowing users to export the complete dataset or extract customized subsets based on specific criteria, facilitating offline analysis and integration with local workflows.

### Case study

**Fig. 5:**
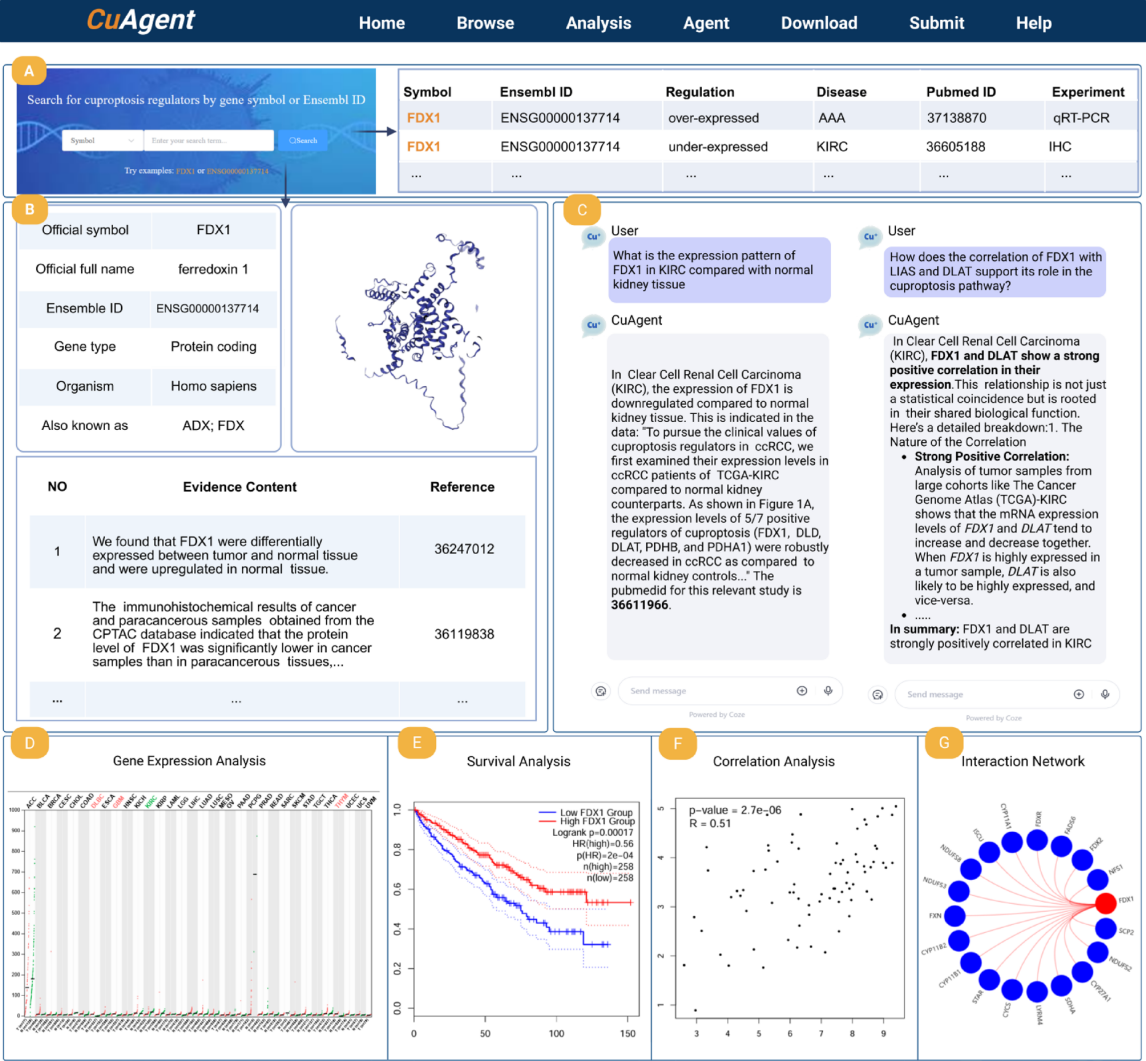
Gene detail page for “FDX1”: an application case study. (**A**) Data retrieval. (**B**) Gene information integration. (**C**) Intelligent Q&A agent. (**D**) Gene expression. (**E**) Survival analysis. (**F**) Correlation analysis. (**G**) Interaction network.

To demonstrate the comprehensive functionality of CuAgent in facilitating cuproptosis research, we conducted an integrated analysis of *FDX1*, a pivotal regulator of cuproptosis. A query for “*FDX1*” retrieved 74 experimentally supported associations across 47 diseases, curated from 47 published studies (Fig. 5A). The gene detail page (Fig. 5B) integrates multi-dimensional information including gene identifiers, structural visualization of the [2Fe-2 S] cluster domain predicted by AlphaFold, functional annotations, and experimental evidence from diseases such as kidney renal clear cell carcinoma and thyroid cancer. This centralized integration provides not only a rapid overview of *FDX1* but also a solid foundation for systematic downstream analyses.

KIRC is the most common histological subtype of renal cell carcinoma, accounting for approximately 70–80% of cases. KIRC is characterized by distinct metabolic reprogramming, particularly alterations in mitochondrial function and energy metabolism, which makes it a highly relevant context for investigating cuproptosis-related genes. Therefore, our subsequent biological analyses are also based on the KIRC dataset.

In KIRC, our expression analysis demonstrated that *FDX1* is significantly down-expressed in tumor tissues compared with normal kidney tissues (Fig. 5D). This observation is consistent with published evidence. For example, Using immunohistochemistry (IHC), Yao et al.^33^ found that *FDX1 was* lowly expressed in renal clear cell carcinoma, and its low expression was closely associated with poor prognosis and changes in the immune microenvironment. These consistent results highlight *FDX1* down-expressed as a robust molecular feature of KIRC.

We next evaluated the relationship between *FDX1* and *DLAT*, another essential component of the pyruvate dehydrogenase complex, and found a strong positive correlation between their expression levels in KIRC (*R* = 0.64, *p* < 2.2e-16) (Fig. 5F). Experimental evidence from the literature is consistent with these observations. Xie et al.^7^ reported that “the expression levels of *FDX1* and *DLAT* were robustly decreased in KIRC as compared to normal kidney controls,” and further confirmed reduced *FDX1* expression by IHC staining of a KIRC tissue microarray (27 paired tumor/adjacent samples; *n* = 27). In addition, Jiang et al.^34^ demonstrated the tumor-suppressive function of *DLAT* by constructing a *DLAT*-overexpressing lentivirus and performing CCK-8 proliferation assays (overexpression significantly inhibited proliferation of KIRC cell lines, *p* < 0.001), Transwell migration assays (significant reduction in migration, *p* < 0.001), and in vivo xenograft experiments (reduced tumor volume and weight, *p* < 0.01). Together, the reported IHC evidence of co-downregulation and the functional assays confirming *DLAT’s* tumor-suppressive activity are concordant with our correlation analysis and suggest a potential functional interdependence of *FDX1* and *DLAT* within the cuproptosis pathway in KIRC. Together, the reported IHC evidence of co-downregulation and the functional assays not only confirm the high correlation between *DLAT* and *FDX1* expression but also suggest their potential functional interplay within the cuproptosis pathway in KIRC.

Finally, Kaplan–Meier survival analysis indicated that high *FDX1* expression predicts significantly better overall survival in KIRC patients (HR = 0.56, log-rank *p* = 0.00017) (Fig. 5E). This agrees with prior studies, for example, Yao et al.^33^ performed a tissue microarray analysis by IHC using paraffin-embedded paired tissues from 199 patients. Patients were divided into high and low *FDX1* expression groups according to IHC scores, with scores of 4 or less assigned to the low expression group, revealed that patients with relatively low *FDX1* expression were more frequently associated with advanced stages, including higher AJCC stage, WHO/ISUP grade, and T and M stages (*p* < 0.05 for all comparisons). In addition, we explored the protein–protein interaction (PPI) network of *FDX1* to investigate its functional interactions at the protein level. Using a correlation score threshold of 85, *FDX1* was predicted to interact with multiple mitochondrial and metabolic proteins (Fig. 5G). Collectively, the biological analyses offer a multi-layered view of *FDX1*, integrating transcriptional expression, survival relevance, correlation with pathway genes, and protein interaction partners, thus providing a comprehensive basis for mechanistic investigation and translational research.

The intelligent Q&A agent recapitulated these results in real time: when asked about expression or correlation patterns, it returned evidence-based explanations consistent with published data, while also providing mechanistic and clinical context (Fig. 5C).

Importantly, the Q&A agent contextualized this relationship within a clinical framework: Lower expression of *FDX1* and *DLAT* is associated with advanced tumor stage and poorer survival, whereas restoration of their activity could represent a therapeutic opportunity by reactivating the cuproptosis pathway.

These examples illustrate how the intelligent Q&A agent complements quantitative analyses by not only reproducing established statistical relationships but also providing mechanistic and clinical context directly linked to curated literature evidence. This ensures transparency, reproducibility, and facilitates rapid hypothesis generation.

Compared with biological analyses, which require extensive datasets and statistical validation, the agent achieves rapid knowledge retrieval and mechanism-oriented interpretation in seconds, with all responses grounded in curated experimental evidence. While biological analyses delineate quantitative associations and clinical relevance, the agent complements them by providing mechanistic insights and contextual explanations directly supported by the literature. This synergy between data-driven analysis and intelligent question answering highlights the unique advantage of CuAgent: it not only supports the systematic dissection of cuproptosis-related genes through computational analysis but also accelerates scientific reasoning and hypothesis generation through interactive, literature-based knowledge integration.

## Conclusion

CuAgent introduces a novel approach to the acquisition and processing of biomedical knowledge. By integrating an AI-assisted natural language processing engine, CuAgent enables users to query complex biological questions and obtain evidence-based answers in real time, significantly accelerating the hypothesis generation and experimental design process. Compared to traditional database querying methods, CuAgent provides a more flexible and efficient interactive query platform, allowing researchers to quickly extract and analyze relevant information according to their specific needs, thereby significantly improving data utilization efficiency.

A key feature of CuAgent is its knowledge base built through large-scale literature mining. By systematically analyzing and extracting the latest scientific literature, CuAgent integrates a vast amount of biomedical information, including gene data, experimental results and clinical observations. The platform relies on extensive literature resources, but rigorous selection and validation mechanisms ensure that the extracted data is of high reliability. During the literature mining process, the platform prioritizes high-quality peer-reviewed journal articles and filters data based on consistency and reliability. Additionally, CuAgent continuously updates its dataset to maintain the timeliness and accuracy of its knowledge base.

In terms of data analysis, CuAgent provides several conventional bioinformatics analysis tools, including gene expression analysis, survival analysis and the construction of PPI network. These analytical functions not only help researchers gain deeper insights into the data but also provide strong support for hypothesis generation. By performing in-depth analysis of CRGs and their biological functions, the platform reveals potential relationships between different genes and the copper death mechanism, helping researchers identify new therapeutic targets. CuAgent also integrates these analytical results with user queries, providing a basis for personalized experimental design and data interpretation.

The most innovative aspect of CuAgent is its AI-assisted interactive query system. Through natural language processing technology, CuAgent not only provides standard database queries but also simulates interactive dialogue with the user. Researchers can ask questions using simple natural language input, and the platform provides immediate and evidence-based answers based on data and literature extracted from its knowledge base. This intelligent dialogue feature greatly simplifies complex query processes, as researchers no longer need to manually sift through data or integrate information. Instead, they can quickly obtain relevant insights and research recommendations. The application of AI makes CuAgent not just a static query tool but a dynamic system that interacts with users and provides personalized answers based on their needs.

In summary, CuAgent offers a new way of data acquisition and analysis for biomedical research by combining large-scale literature mining, conventional bioinformatics analysis tools and advanced AI-assisted interactive functions. By ensuring the reliability and timeliness of data, CuAgent not only provides researchers with a powerful tool but also lowers the barrier to accessing information and enhances the efficiency of the research process through its interactive AI module. As the platform continues to expand and optimize, CuAgent’s applications in cuproptosis research and other biomedical fields are expected to grow significantly.

Taken together, while CuAgent offers a robust interactive framework, it is fundamentally designed as an evidence-based auxiliary tool rather than a de novo systems biology model. Its integrated bioinformatics modules are standard analytical tools intended solely to provide a quantitative context for text mining. To strictly mitigate the risk of AI hallucinations, we deliberately excluded automated causal inference, restricting the platform to the precise retrieval of experimentally validated research. Ultimately, CuAgent lowers the barrier to complex information retrieval, empowering researchers to leverage reliable, literature-backed clues for their own independent causal interpretation and hypothesis generation. To ensure the database remains current, the CuAgent knowledge base will be systematically updated every six months to incorporate newly validated experimental findings. All modifications will be detailed in an update log on the platform’s homepage.

## Data Availability

CuAgent and data information is available at https://bioinfor.nefu.edu.cn/CuAgent/home/.
